# Just choice: a Danielsian analysis of the aims and scope of prenatal screening for fetal abnormalities

**DOI:** 10.1007/s11019-019-09888-5

**Published:** 2019-02-15

**Authors:** Greg Stapleton, Wybo Dondorp, Peter Schröder-Bäck, Guido de Wert

**Affiliations:** 1 Department of Health, Ethics and Society, GROW School for Oncology and Developmental Biology, Faculty of Health, Medicine and Life Sciences, Maastricht University, 6200 MD Maastricht, The Netherlands; 2 Department of International Health, CAPHRI Care and Public Health Research Institute, Faculty of Health, Medicine and Life Sciences, Maastricht University, Maastricht, The Netherlands

**Keywords:** Ethics, Justice, Non-invasive prenatal testing, Prenatal screening, Public health, Reproductive choice

## Abstract

Developments in Non-Invasive Prenatal Testing (NIPT) and cell-free fetal DNA analysis raise the possibility that antenatal services may soon be able to support couples in non-invasively testing for, and diagnosing, an unprecedented range of genetic disorders and traits coded within their unborn child’s genome. Inevitably, this has prompted debate within the bioethics literature about what screening options should be offered to couples for the purpose of reproductive choice. In relation to this problem, the European Society of Human Genetics (ESHG) and American Society of Human Genetics (ASHG) tentatively recommend that any expansion of this type of screening, as facilitated by NIPT, should be limited to serious congenital and childhood disorders. In support of this recommendation, the ESHG and ASHG cite considerations of distribution justice. Notably, however, an account of justice in the organization and provision of this type of screening which might substantiate this recommendation has yet to be developed. This paper attempts to redress this oversight through an investigation of Norman Daniels’ theory of Just health: meeting health needs fairly. In line with this aim, the paper examines what special moral importance (for Just health) screening for the purpose of reproductive choice might have where concerning serious congenital and childhood disorders in particular. The paper concludes that screening for reproductive choice where concerning serious congenital and childhood disorders may be important for providing women with fair opportunity to protect their health (by either having or not having an affected child).

## Introduction

Public health services within many western countries routinely offer pregnant women the option to participate in some type of screening programme. Generally speaking, women are most routinely offered the opportunity to screen for a number of preventable infectious diseases (e.g. HIV, Syphilis, Hepatitis B, and asymptomatic bacteriuria) and clinical conditions (e.g. gestational diabetes, pre-eclampsia, anemia, and rhesus D incompatibility). For these conditions, early (prenatal) detection of disease generally provides affected women with timely access to preventative healthcare interventions that protect the health of their (future) child. Similarly, the early detection of disease may also provide affected women with access to preventative treatments that protect their own health (HCN 2008). Since for these conditions, screening carries obvious health benefits for women and their children, there is relatively little controversy about organizing its provision within a public health framework that is aimed at (collective) prevention (HCN 2008).

However, public health services within many countries also routinely offer women a different type of screening option. Unlike screening for infectious diseases and clinical conditions, screening for fetal abnormalities is different in that it is not offered in order to prevent the targeted conditions from affecting the child. For many conditions that are detected through this type of screening, reliable options for preventative healthcare are unavailable (i.e. not possible). In cases where they are, their use may only facilitate relatively limited improvements in health outcomes for the affected child. The situation is further complicated by the fact that many of the follow-up interventions that are available may also involve the use of invasive procedures that carry maternal health risks (Danzer et al. 2012; Farmer 2003; Häger et al. 2004; Sutton 2008). In these cases, women are often left with few options other than to make a reproductive decision about whether or not to end the pregnancy. In view of the historical eugenic connotations associated with offering this type of screening, international guidelines now recommend that screening for fetal abnormalities is only morally defensible when offered within a non-directive framework that facilitates couples in making *meaningful* reproductive choices; typically qualified as relating to informed and autonomous reproductive decisions about having or not having an affected child that enable couples to avoid suffering they anticipate for themselves and/or their future child (de Jong and de Wert 2015; Dondorp et al. 2015; HCN 2008).

At present, this type of screening (screening for reproductive choice) is most routinely offered for a limited range of serious congenital disorders; namely, chromosomal abnormalities such as Down’s syndrome, Edward’s syndrome, and Patau’s syndrome, structural malformations such as anencephaly, spina bifida, serious cardiac abnormalities, diaphragamtaic hernia, gastroschisis, exomphalos, bilateral renal agenesis, lethal skeletal dysplasia, and for recessive monogenetic conditions such as the haemoglobinopathies sickle-cell and thalassaemias (Boyd et al. 2008; HCN 2008; NCCWCH 2008). Up until recently, available techniques for prenatal testing have provided women with relatively little opportunity to screen for conditions that fall beyond this limited range. However, the situation is now changing. Developments in non-invasive prenatal testing of maternal serum (NIPT) and genetic analysis of cell-free fetal nucleic acid (cff-DNA) are raising the prospect that antenatal services will soon be able to provide women with the option of screening for an unprecedented range of genetic conditions and traits (Hui and Bianchi 2017; Wong et al. 2016). Predictably, such developments are now prompting considerable debate within the bioethics literature about the possibility of expansion; more specifically, about what conditions should be prioritized when offering screening for a broader range of conditions would be irresponsible in light of resource constraints (Allyse et al. 2017; Berkman and Bayefsky 2017; Botkin et al. 2017; Chen and Wasserman 2017; Conley II et al. 2017; de Jong and de Wert 2015; Munthe 2015; Stapleton 2017; Wilkinson 2015).

In relation to the issue, the European Society of Human Genetics (ESHG) and American Society of Human Genetics (ASHG) recommend that, pending further discussion, any expansion in the scope of prenatal screening for fetal abnormalities should be limited to serious congenital and childhood disorders (Dondorp et al. 2015). In their joint statement, they list several arguments in support of this recommendation, including concerns about information overload, about undermining the autonomy interests of any children that might be born after a positive finding, about the trivialization of abortion related to testing for non-health related characteristics, and about limit-setting in light of considerations of distributive justice. Whereas the former three concerns have been critically discussed within ongoing debates in the bioethics literature, there has not until now been much critical discussion about the concern relating to distributive justice (Dondorp et al. 2015; Stapleton 2017). On this issue, the two societies suggest that where prenatal screening is funded as a public health service, resource constraints may require limit-setting (in line with the proposed framework) as a matter of distributive justice. In this paper we explore this idea by investigating how an established theory of justice in health, healthcare, and public health might be applied to the organization and provision of prenatal screening for fetal abnormalities.

Inevitably, this requires us to consider how debates about the aims of offering prenatal screening for serious congenital and childhood disorders might relate to healthcare and public health objectives that are established within theories of justice and health. Notably, that there may be a tension here is highlighted by the ESHG and ASHG in their joint statement: *“When prenatal screening for fetal abnormalities is publicly funded, considerations of distributive justice point in the same direction [to prioritizing serious congenital and childhood disorders]. Even when, with decreasing sequencing costs, it will become possible to chart the full genome of the fetus in one test, it will still be the case that a wider range of possible outcomes will require more information and more complex counseling. Inevitably, this requires defining ‘meaningful reproductive choices’ in a way that can be recognized by the tax payers whose solidarity is invoked to uphold the service, rather than leaving this to the private understanding of the pregnant woman and her partner*.” (2015, p. 8). Whilst making the point that, within a public health framework, limit-setting for the sake of justice may be unavoidable, the two societies suggest that doing so may be at odds with the idea that prenatal screening for serious congenital and childhood disorders is offered to provide women (and their partners) with the opportunity to make reproductive choices *that are meaningful to them* (Stapleton 2017).

At present, a suitable public health framework for *selectively* funding prenatal screening to facilitate meaningful reproductive choice—where concerning serious congenital and childhood disorders in particular—has yet to be agreed upon. In this paper we attempt to address this oversight. To achieve this, we extend Norman Daniels’ widely acclaimed theory of justice in health, healthcare, and public health—*Just health: meeting health needs fairly*—to the organization and provision of prenatal screening services (2008). In the first part of the paper, an introductory account of Daniels’ main thesis is provided. In this section, the special moral importance for justice of health, and thus of healthcare and public health services, is discussed. In the second section of the paper, Daniels’ theory is applied to the organization and provision of different types of prenatal screening. Here, the utility of prenatal screening for different types of conditions is assessed with respect to promoting the healthcare and public health objectives established within *Just health*. Special attention is then given to the analysis of prenatal screening for fetal abnormalities and to the offer of reproductive choice. The main conclusions emerging from this analysis are then discussed in relation to the ESHG and ASHG recommendations concerning the scope of prenatal screening for fetal abnormalities.

## Justice in health, healthcare, and public health

Where concerning questions of justice in the organization of healthcare and public health services, many theorists hold to the ideas of Norman Daniels (1981, 2001, 2008). Within his widely acclaimed theory of *Just Health: meeting health needs fairly*, Daniels ascribes moral value to health on the basis of its contribution towards protecting the normal range of *opportunities* that are open to all within society. As noted by Daniels, many contemporary theories of justice are consistent with the idea that justice holds to some principle of *equality of opportunity*. Whilst it is relatively intuitive what the principle requires in terms of the protection of liberties and freedoms, it is less intuitive what it requires in the distribution of opportunity-affecting resources. In relation to this point, competing views on justice generally converge on the idea that goods and services should be organized in such a way that they compensate individuals for any *unfair* loss of such resources (those that are beyond individual control) (Rawls 1971, 2001; Roemer 1996). From this perspective, a just distribution of resources should meet the criteria that it assures all individuals a normal range of opportunities within society. In line with this idea, Daniels reasons that since health helps protect the normal range of opportunities that should be assured for all in society, protecting health is important for justice (2008). In the following section we provide an introductory account of the main ideas Daniels presents in support of this position.

### What is the special moral importance of health?

Within *Just health*, Daniels conceptualizes health as an objectively ascribable good that normally enables individuals to pursue opportunities within society. In this way, he suggests that protecting health contributes towards equality of opportunity. Drawing upon a Boorsian model of health, Daniels considers health in terms of statistical normality of species typical functions (those which contribute towards species typical aims of survival and reproduction) as controlled by sex, age, and environment (Boorse 1975, 1977, 1997; Daniels 2008). Illness, disease, and disability are represented by deviations in ‘normal functioning’ that are detrimental to the species typical objectives of survival and reproduction. Daniels argues that harmful deviations in ‘normal functioning’ are especially problematic from the perspective of justice as they limit one’s ability to compete for positions and offices within society (2008). Since healthcare, and other goods and services that protect health (as normal functioning) also protect opportunity, access to these goods and services may also be seen as important for justice.

Criticisms of this account of justice may be directed at the choice of normative criteria that are used to ascribe moral importance to ‘normal functioning’. From a welfare-based conception of the good, socio-economic prospects are only important to the extent that they contribute towards individual well-being and happiness. Accordingly, the benefit that ‘normal functioning’ offers in terms of maintaining an individual’s socio-economic prospects is considered derivative. Instead, promoting well-being should be the principle focus of distributive justice. In line with this idea, the moral importance of various goods and services should be evaluated according to the strength and urgency of an individual’s (subjective) preferences. Distributive justice may then be realized within something like a free market where all individuals are assured fair opportunity to invest according to their preferences (Arneson 1989; Daniels 2008; Scanlon 1975).

Daniels offers two main counter arguments to such criticisms. First, he suggests that protecting normal functioning has a definite tendency to promote individual well-being. Yet, it also protects the normal range of exercisable opportunities that are open to individuals from which to pursue their own conception of the good. Thus, since normal functioning contributes both to protecting well-being directly and to protecting an individual’s opportunities for well-being, then protecting health as normal functioning may also be valued for protecting well-being (2008). Second, he argues that distributing resources according to preferences is problematic with respect to considerations of fairness. Following such an approach, resources may be distributed in a way that is insensitive to considerations of brute luck and moral desert. Considerations of brute luck and moral desert are concerned with what each individual deserves from one another in society. They relate to the idea that some individuals have greater need than others, and thus, are more deserving of assistance. Evaluative frameworks that ascribe moral importance to the distribution of resources on the basis of preference-satisfaction do not account for such differences, i.e. they are unfair. Using such an approach, individual preferences may be valued more or less equally, irrespective of whether they are urgent and cheap, or superficial and expensive (Daniels 2008).

Relating to this point, Daniels argues that an evaluative framework that focuses on *meeting needs*, rather than *satisfying preferences*, will facilitate a fairer distribution of resources. As Daniels points out “*needs and preferences are not equivalent*” (2008, p. 31). Needs are those things that are important to people whether or not they are always aware of them. People may not always be conscious of them, yet, with hindsight, know when they have not been met. In this respect, they are often more urgently coveted when left unfulfilled. From a welfare perspective, needs refer to those goods that, without, cause suffering. As such, they are prerequisite for well-being. In contrast, preferences are valued more subjectively. Although satisfying preferences may contribute towards well-being, it is not essential for maintaining it. Building upon this general idea, Daniels argues that meeting needs is inherently more important for promoting individual well-being than satisfying preferences.

Daniels points out that free market approaches, which presume to offer individuals *fair opportunity* for resources, overlooks the fact that an objective framework for evaluating what constitutes *fair opportunity* is still required in order to account for pre-existing inequalities which confer varying levels of need. Thus, an agreement on a suitable framework for evaluating need is still required. Daniels suggests that this may be overcome by appealing to fair procedure in which overlapping consensus between reasonable people is used to decide upon a suitable framework. He goes on to argue that such a consensus is most likely to be achieved where concerning a naturalist framework of health as normal (species typical) functioning (2008). *Health needs* may then be assessed and compared with reasonable objectivity using the biomedical sciences in order to facilitate the provision of healthcare (in line with meeting health needs).

### What are the main responsibilities of public health services?

Whilst Daniels’ account of justice provides a relatively uncontroversial explanation for why healthcare is important for justice (when it meets health needs), it does not provide an explanation of when (the rest of) society might be obliged to pay for it. To address this issue, he investigates what might make inequalities in health unjust, and therefore, evoke obligations of financial support. Regarding this issue, Daniels concludes that inequalities in health may be considered unjust when they violate established principles of equality of opportunity. He illustrates this point using principles of justice taken from John Rawls’ landmark theory of *Justice as fairness*: “*The general principles of justice that Rawls argues for in his theory of justice as fairness capture the key social determinants of health, especially if one includes, as I do, health care in the institutions that protect opportunity*.” (Daniels 2008, p. 23; Rawls 1971, 2001).

Within *Justice as fairness*, Rawls takes the position that a just system of social and economic cooperation will invariably hold to two basic principles: “*(a) Each person has the same indefeasible claim to a fully adequate scheme of equal basic liberties, which scheme is compatible with the same scheme of liberties for all; and (b) Social and economic inequalities are to satisfy two conditions: first, they are to be attached to offices and positions open to all under conditions of fair equality of opportunity; and second, they are to be to the greatest benefit of the least-advantaged members of society (the difference principle).*” (Rawls 2001, p. 42–43). The first principle mainly emphasizes (negative) obligations of respect for persons (i.e. of non-interference); individuals should not be victimized through discrimination when exercising self-determination within a basic scheme of liberties (Buchanan 1995; Buchanan et al. 2001). The second principle (the principle of *fair equality of opportunity* and the qualifying *difference principle*) provides a basic framework for regulating the distribution of socio-economic goods within society.

According to the principle of *fair equality of opportunity*, individuals with similar talents and abilities should expect to have similar socio-economic prospects. They should not be awarded on the basis of morally arbitrary factors such as sex, race, or socio-economic status (characteristics that an individual has no control over and are not relevant to the work advertised). The negative force associated with this obligation is connected to ideals of non-discrimination that are set by the first principle. Up until this point, it has been assumed that socio-economic endowments are distributed, more or less, equally; in other words, justice has thus far been conceived within a society where the inheritance of socio-economic endowments among similarly abled people is unaffected by *brute luck*. However, when this assumption is removed, the second principle evokes (positive) obligations towards resource redress in line with compensating individuals for any opportunity-loss resulting from bad brute luck in this type of inheritance. These obligations may be derived from the qualifying difference principle; which aims to regulate the distribution of lifetime prospects among individuals in society (assessed by an index of ‘primary social goods’) (Daniels 2008; Rawls 1971, 2001). The difference principle demands that any inequalities in socio-economic prospects should meet the requirement that they act to the benefit of all within society, and most importantly, maximally benefit its least advantaged members.

Critically, the above specification of Rawlsian principles provides no justification for resource redress where concerning bad luck in the natural endowments one inherits (e.g. innate talent and natural ability and/or illness, disease, and disability). In developing the two principles of justice, Rawls (famously) avoids discussing health and disability, electing instead to develop his theory of justice within an ideal (hypothetical) context in which he maintains the crude assumption that all members of society are otherwise healthy and normally functioning (Rawls 1971, 2001). It is primarily in relation to this assumption that Daniels has developed his theory of *Just health* (Daniels 1981, 2001, 2008). As noted by Daniels, the main justifications put forward by Rawls for eliminating opportunity-loss resulting from undeserved deficits in socio-economic endowments (e.g. inherited wealth) also seem to apply where concerning the opportunity-limiting effect of underserved deficits in natural endowments (e.g. inherited health). Accordingly, if resource redress for socio-economic disadvantage is important because it promotes fair equality of opportunity, it follows that access to healthcare is also important whenever it contributes towards the same end. On this basis Daniels includes within the scope of the Rawlsian principle of fair equality of opportunity an obligation towards maintaining a fair distribution of socially controllable determinants of health; including socio-economic goods that might affect healthcare access (e.g. income). As Daniels points out, this interpretation establishes a basic imperative for the organization of public health services (2008).

## Prenatal screening and Just health

Daniels concludes that health has special moral importance for justice because it protects the normal range of opportunities that should be assured for all according to widely accepted principles of equality of opportunity. In line with this idea, Daniels suggests the following: (i) healthcare services should be aimed *at restoring and protecting health*, defined in terms of statistical normality of species typical functions (those which contribute towards species typical goals of survival and reproduction) controlled by sex, age, and environment; (ii) that public health services should aim *to maintain a fair distribution of socially controllable determinants of health* (e.g. access to goods and services that protect health). With respect to the latter point, Daniels suggest that what constitutes a fair distribution of socially controllable determinants of health may be determined using (for example) Rawlsian principles of fair equality of opportunity (Daniels 2008; Rawls 1971, 2001). According to this interpretation, it is possible to infer that offering screening for preventable conditions is important for *Just health* whenever it provides affected individuals with timely access to preventative healthcare (that protects their health as normal functioning). Since in these cases screening is a prerequisite for accessing preventative healthcare, and since access to screening services is socially controllable, then the provision of screening will fall within the scope of the public health objectives established within *Just health*. In other words, when the use of screening is important for accessing preventative healthcare, its provision can help maintain a fair distribution of socially controllable determinants of health among participants, and thus, contribute towards promoting *Just health*.

This account of *Just health* is relatively consistency with public health policy where concerning prenatal screening for infectious diseases (HIV, Syphilis, and Hepatitis B) and for clinical conditions (gestational diabetes, pre-eclampsia, anemia, and rhesus D incompatibility). In these cases, screening facilitates the detection of preventable health risks that without timely intervention may seriously impact the health of future children. It enables women to access interventions for primary prevention that significantly reduce the risk of any mother-to-child perinatal-transmission of infection and/or injury. Promoting women’s access to screening may thus contribute towards maintaining a fair distribution of socially controllable determinants of health among future children. Notably, however, as women may also benefit from more timely access to interventions for secondary prevention, facilitating them in accessing the screening service may also be relevant in maintaining a fair distribution of socially controllable determinants of health among women. Given the favourable public health outcomes for both women and future children, it is relatively evident that, if public health services aspire to promote *Just health*, then they should routinely offer women screening for these conditions. However, the situation is somewhat different where concerning screening for fetal abnormalities. For these conditions, screening may not so effectively enable women to protect the health of their future child or to protect the their own health. For example, primary prevention for an affected fetus is generally not possible at the point of screening. Yet in many cases, interventions for secondary prevention are also unavailable. As such women may be left with few practical courses of action other than to decide whether or not to continue with the pregnancy.

Additionally, in cases where interventions for secondary prevention are available, they may involve the use of invasive surgical procedures that carry with them significant maternal health risks. For example, in the case of myelomeningocele, the option of in utero surgical repair is possible following early (prenatal) diagnosis. In this instance, the intervention can improve developmental outcomes for the child. However, the procedure carries a risk of fetal loss and may also cause women the following health complications: Surgical bleeding, infection, preterm rupture of membranes, preterm labor and delivery, medication and anesthesia complications, prolonged hospitalization, and need for delivery by Cesarean section for the current pregnancy and any future pregnancies (Danzer et al. 2012; Farmer 2003). The situation is somewhat similar for interventions that are offered in a broader variety of fetal abnormalities. For example, delivery by Cesarean section may be indicated for certain structural malformations. This can reduce the risk of injury to the fetus from vaginal delivery. Although the risk of fetal loss is less relevant in this instance, the procedure may still cause women a range of significant health complications (Anteby and Yagel 2003; Hadar et al. 2011; Häger et al. 2004; Sutton 2008; Villar et al. 2007; Wataganara et al. 2017). In view of these issues, it remains unclear whether routinely offering women prenatal screening for fetal abnormalities would not in fact detract from the pursuit of *Just health*.

Two issues appear to require further investigation. First, what are the main (health) benefits received by women from the use of follow-up interventions that aim to protect the health of their future child (i.e. where interventions for secondary prevention are available)? How might the proportionality of these benefits be assessed in light of the maternal health risks that such interventions can sometimes carry? Second, in cases where interventions for preventative healthcare offer little protection to the future child or are unavailable altogether, what (health) benefits might women derive from the remaining option of reproductive choice? How might this benefit be evaluated? In the following section we address these issues through a detailed examination of the health benefits women may receive (in terms of normal functioning) following the use of follow-up interventions that aim to (i) protect the health of their future child and (ii) facilitate women in making reproductive choices.

### Screening to protect the health of the future child

Daniels’ framework of health as normal functioning offers two main normative criteria for evaluating the maternal health benefits associated with screening for fetal abnormalities. Within *Just health*, normal functioning is evaluated according to species typical aims of both ‘survival’ and ‘reproduction’ (Boorse 1977; Daniels 2008). With respect to the latter criterion, any deviation in bio-statistical normality of physiological functioning between a reproductive couple that might detract from having a healthy or normally functioning child would seem, at least conceptually speaking, representative of a *reproductive* illness, disease, or disability. From this perspective, prenatal interventions that are aimed at protecting the health of the future child may also be valued as protecting women’s *reproductive health* or *normal reproductive functioning*. Notably, this raises the prospect that any health risks incurred by women from the use of follow-up interventions may be offset by improvements achieved in health outcomes for the future child; or in terms of a woman’s ‘reproductive health’. With respect to this point, an important question is raised about how much importance should be placed on protecting this type of maternal health given that women may incur risks to other aspects of their physical health.

Unlike with physiological functions that contribute towards species typical aims of ‘survival’, many of those that contribute towards the alternative aim of ‘reproduction’ are different in the sense that they are not always needed. In this way, they are somewhat secondary to those that contribute towards survival. For the majority of us, there are perhaps relatively few moments in our lives when we do not consider our survival a priority, and far fewer that we intentionally try to avoid it. By contrast, reproduction (as in having children) is something that throughout one’s life is more readily than not consciously avoided. It is a more complex need in that it is something that one may only feel strongly about during a few, very specific times throughout their life. For the remainder, what one most readily needs is control over their fertility; i.e. the ability to choose when and when not to have children. When this ‘need’ is unmet, it causes suffering. In this respect, an individual’s (reproductive) choices are very important in discerning their reproductive needs, and thus, for discerning their overall health needs. Critically, however, Daniels’ framework of health as normal functioning has been (expressly) developed in order to avoid having to take individual choice into account (2008). As a result, there are some challenges in using the framework to comparably evaluate the relative importance of protecting either reproductive or non-reproductive aspects of a women’s health.

First, where women incur a risk to aspects of their physical health during pregnancy, the framework of normal functioning must take into account each woman’s subjective evaluation of the relative importance of the health outcomes that are available to her. Although most individuals may prioritize protecting aspects of their health that contribute towards their survival, the relative importance of pursuing this goal may vary significantly between women pending the severity of the risk to their health and the strength of their reproductive needs. As such, different women may feel strongly about varying levels of compromise. Yet, the framework of normal physical functioning does not provide any criteria that might account for this subjectivity. Second, this problem can be further complicated by the fact that interventions used to protect a woman’s reproductive health may also undermine other aspects of her reproductive functioning. For example, interventions that are offered to improve health outcomes for the expected child may compromise a woman’s ability to have (other) children in the future. In such instances, a woman’s reproductive needs might appear conflicting. However, the framework of normal physical functioning does not provide any criteria that could be used to account for this. This raises the question: what might be suitable criteria for evaluating a woman’s health needs in light of such conflicts?

### The importance of protecting women’s mental health

Regarding this issue, it is relevant to note that within *Just health*, ‘health’ is not evaluated exclusively in terms the ‘physical’. Daniels states that: “*I understand health to mean normal functioning – the absence of significant mental or physical pathology*” (Daniels 2008, p. 2). In this respect, *Just health* also demands protecting women’s ‘mental’ health. In earlier work, Sabin and Daniels clarify how normal functioning might be understood in terms of mental health: “*The normal functioning model takes unequal distribution of human capabilities as fact that health care will not change. Some people are socially adept. Others are shy in ways that cause suffering. The model prescribes compassion for those who are less fortunate in the natural lottery that distributes capabilities, but makes the health sector responsible for correcting only those conditions which – in DSM-IV terms – can be diagnosed as* ‘*a symptom of a dysfunction*’, *that is, as mental disorders*.” (Sabin and Daniels 1994, p. 10). Following this idea, it would seem reasonable to infer that the relative importance of the different health outcomes available to each woman may be comparably assessed using measures of *mental health*. For example, where the use of an intervention for protecting the health of the future child also conveys a demonstrable improvement in women’s mental health, it would seem reasonable to infer that the use of the intervention (as opposed to avoiding its use) is important for meeting women’s health needs. It then follows that routinely offering women screening for the relevant condition would also positively contribute towards promoting *Just health* among women.

Notably, concerns about the mental health of women with an affected pregnancy appear well-justified (Fonseca et al. 2014; Golfenshtein et al. 2016a, b; Korenromp et al. 2005a, b, 2009). Studies into psychological distress among different parent groups demonstrate that having a child with a serious congenital disorder is associated with increased levels of psychological distress and reduced quality of life (Lawoko et al. 2002, 2003; Statham et al. 2000). Related studies into the psychological adjustment of parents of affected children also indicate some association between the level of distress that is experienced and the severity of the congenital disorder that has been diagnosed (Brosig et al. 2007; Fonseca et al. 2012, 2014). Thus, there already appears at least some cursory evidence to suggest interventions that can improve health outcomes for the future child may also be useful in mitigating the psychological distress that is associated with parenting an affected child. If future empirical study substantiates this claim, it would lend support to the idea that screening to protect the health of the future child would also be beneficial for protecting women’s health.

### Screening for reproductive choice

Critically, considerable empirical evidence also suggests that the diagnosis of an *untreatable* congenital disorder is itself a significant cause of psychological distress among affected couples (Fonseca et al. 2012, 2014; Statham et al. 2000). Taking into consideration the fact that, for many fetal abnormalities, preventative interventions are unavailable, it may be the case that for many women screening for fetal abnormalities would only act to detract from their mental health during the course of pregnancy (Hall et al. 2000; Statham 2000). In the respect that this may only exacerbate the health risks that women might incur, one may easily interpret the onus on services to point in the direction of limiting women’s access. As Statham et al. point out: “*When parents have no choice but to continue the pregnancy, the question that must be asked concerns how they evaluate foreknowledge. Presumptions are made that there is the opportunity for emotional preparation, but there is no empirical evidence as to whether this is psychologically helpful for women in general, […]*.” (2000, p. 734). It follows accordingly that routinely offering women this type of screening would not be especially important in meeting their health needs.

However, when women receive the option of terminating an affected pregnancy, a number of additional considerations are relevant to discuss. In relation to this point, it is important to first note that, historically speaking, screening for fetal abnormalities was readily offered for the purpose of reducing the birth rate of children with serious congenital disorders (such as Down’s syndrome). Within public health policy recommendations published on behalf of the Health services and mental health administration in the US during the early 1970s, prenatal screening and selective abortion are explicitly valued for ‘preventing’ Down’s syndrome: “*The rising expectation of life for persons with Down’s syndrome makes it all the more important to focus on prevention. [...] We advocate four specific preventive measures to reduce the incidence of Down’s syndrome at birth, namely, education, birth control, prenatal diagnostic testing, and elective termination of pregnancy*.” (Stein and Susser 1971, p. 657). In line with this idea, clinical practices were often prescriptive. They promoted women’s participation in screening and, following a positive diagnosis, the elective termination of pregnancy. Nadler observed that: “*Since adequate treatment or correction of these disorders has not been available, emphasis has been placed on prevention in the forms of genetic counseling and*/*or therapeutic abortion*.” (Nadler 1969, p. 132).

Such practices have since received wide international condemnation for their negative eugenic connotations (Van El et al. 2012). However, from the perspective of *Just health*, the principle issue with such practices is that they appeal to a (public health) framework in which the direction of solidarity that is normally evoked for reasons of fairness, i.e. between the tax-payer and towards the provision of healthcare for affected families, is reversed. The tax-payer is considered the least advantaged of the two groups; they are burdened by couples choosing to have children with complex health needs that increase the demand on public health services. Implied by this is the idea that couples *should* participate in screening, and upon receiving a positive finding, *should* elect to terminate the pregnancy. Whilst this is intuitively problematic, it also overtly conflicts with the healthcare and public health objectives established within *Just health*. First, implicit in the view that the termination of an affected pregnancy is unproblematic with respect to promoting healthcare and public health objectives established in *Just health*, is the idea that, in the eyes of justice, an affected fetus is not a person and is thus undeserving of any health protection that may be realized through the organization and provision of antenatal services. Yet, this view is already controversial. Although views about the moral status of the fetus are divisive, competing perspectives generally converge on the idea the fetus has (at least) some type of moral standing that should be respected (Aramesh 2007; Gillon 2001; Marquis 1989; Pennings and de Wert 2003; Steinbock 2011). Subsequently, the onus on services for healthcare would be, *prima facie*, towards protecting the health of women and their children, and thus for public health services, towards the provision of services that enable women to have a healthy (normally functioning) child.

Second, taking aside any *prima facie* obligations towards protecting the health of the child, the termination of an affected pregnancy may (independently) be seen as problematic with respect to promoting healthcare objectives among women. Within *Just health*, Daniels considers pregnancy and reproduction to be a normal physiological function: “*When women seek to terminate unwanted pregnancies, whether through a morning-after pill or an abortion, they do not speak of their pregnancy as a disease or a pathological condition. Like the medical professionals who treat them, they view unwanted pregnancies as the result of normal – perhaps all-too-normal – functioning*.” (Daniels 2008, p. 41). This view is consistent with the framework of normal functioning that has been proposed within *Just health* for the purpose of evaluating health; namely, species typical aims of ‘survival’ and ‘reproduction’ (Boorse 1977; Daniels 2008). With respect to the latter criterion, the termination of an affected pregnancy may be considered contrary to the idea of protecting health as normal functioning. In this sense, the onus on services for public health would be, *prima facie*, towards the provision of services that enable women to have a healthy (normal functioning) child, and thus by implication, towards the provision of healthcare services that protect the health of women and their children.

However, situations in which women may incur health complications as a result of having an affected child appear somewhat exceptional. In cases where the health needs of women are at odds with those of the future child, healthcare objectives concerning the future child may give way to those concerning women for reasons of fairness. Where such conflicts arise, screening for reproductive choice may play an important role in enabling women to protect their health without constituting an especially offensive violation of *prima facie* obligations towards the future child. This interpretation of *Just health* is perhaps most intuitive in cases where the seriousness of maternal health risks associated with having an affected child, whether physical or psychological, renders any concerns about protecting the health of the future child more or less immaterial. In situations where health services are unable to assure women the option of having an affected child without jeopardizing their health, it would seem unfair not to offer them the option of reproductive choice. This may be justified on the grounds that it assures women fair opportunity to protect their health, and thus, contributes towards maintaining a fair distribution of socially controllable determinants of health among women. In these cases, the organization and provision of routine prenatal screening would seem relatively consistent with the idea of promoting the public health objectives established within *Just health*.

This interpretation would also seem to hold in cases where the opportunity to have an affected child is available. Although in these cases screening for reproductive choice may have a more significant impact on health outcomes for future children, it may still contribute towards the same public health objective in so far it helps maintain a fair distribution of socially controllable determinants of health among women and future children. In relation to this point, the routine offer of prenatal screening for reproductive choice may contribute most when it is targeted at conditions where (i) women incur *more* serious health risks in order to have an affected child and (ii) health outcomes for the future child are much *poorer* (e.g. in terms of severity, onset, and duration). From this perspective, routinely offering women prenatal screening for reproductive choice, where concerning serious congenital and childhood disorders, would appear relatively congruent with the public health framework established in *Just health*.

### Further considerations of Justice

This interpretation of *Just health* appears most intuitive where screening for reproductive choice is indicated by a high risk to women’s health associated with pregnancy and/or childbirth. In these cases, the contribution that screening for reproductive choice make towards promoting healthcare and public health objectives may be evaluated with relatively little controversy using measures of psychological health. The evaluation of specific follow-up services for either having or for not having an affected child may then be assessed independently using additional measures of women’s health as normal functioning. However, where reproductive choice is indicated by a high risk to women’s health during parenthood, the situation may be more complex. In these cases, two additional considerations of *Just health* are relevant to note. First, where the provision of services for parental support (e.g. paediatric care, counselling, specialist schooling, financial support, etc.) is able to redress health needs among parents of affected children, the provision of these services may weaken any justification for routinely offering women the option of screening for reproductive choice. Notably, this raises a controversial funding issue. Since the need for parental support will only develop after the opportunity for reproductive choice has passed, the justification for offering screening appears somewhat contingent on the strength of guarantee offered by public health services towards the ‘future’ provision of services for parental support. In this respect, considerations of (i) the effectiveness of parental support in meeting the health needs of parents of affected children and (ii) the strength of guarantee as to the future provision of such services, appear salient to evaluating the importance of routinely offering women screening for the purpose of reproductive choice.

Second, in situations where interventions that protect the health of the future child are available during pregnancy which might (indirectly) reduce any health risks associated with parenting an affected child, the imperative to offer screening in order to protect the health of the future child will remain, yet, the imperative to offer screening for reproductive choice will be weakened. In these cases, routinely offering women screening for the purpose of protecting the health of the future child would seem more important (for *Just health*). Accordingly, the organization of a more prescriptive screening service, such as those that are offered for preventable infectious diseases and clinical conditions, may well be preferred for the sake of promoting *Just health*. Notably, however, where a follow-up intervention also carries with it a serious maternal health risk, competing obligations towards protecting the health of women will increase the importance of offering screening for the purpose of reproductive choice. Importantly, in these cases, the evaluation of the screening offer must take into account the availability of preventative interventions and the relative risks women might incur from having an affected child with and without the use of the follow-up intervention.

## Concluding remarks

The investigation into Daniels’ theory of *Just health: meeting health needs fairly* suggests that where women incur a sufficiently serious health risks from having an affected child (with or without intervention), routinely offering women the option of screening for reproductive choice is consistent with promoting the public health objectives established within *Just health*. In these cases, offering screening for the purpose of reproductive choice assures women fair opportunity to protect their health by either having or not having an affected child. Since screening may also benefit the health of the future child (when women elect for this option), it remains compatible with the idea of maintaining a fair distribution of socially controllable determinants of health among both women and future children. In this respect, screening for reproductive choice is likely to contribute more towards the public health objectives established in *Just health* in cases when women are exposed to *more* serious health risks from having an affected child and where health expectations for the child are generally *poorer*. Notably, this interpretation would appear relatively consistent with the joint ESHG and ASHG recommendations concerning the scope of prenatal screening for fetal abnormalities.

Some additional considerations were also noted with respect to evaluating the contribution that screening for reproductive choice might make towards public health objectives established within *Just health*. In cases where offering screening for reproductive choice is indicated by a serious health risk to women during parenthood that may be redressed through the provision of services for parental support, considerations of the effectiveness of these services in redressing this risk, as well as the strength of guarantee into their future provision, appear to be important considerations in the justification of routinely offering women prenatal screening for reproductive choice. With respect to this issue, evaluations may be complicated where prenatal interventions for protecting the health of the child are available. In these cases, the availability of the intervention, and the relative risks women incur from having an affected child (with and without the intervention), would also appear important to consider.
